# Developing a tool for obtaining maternal skinfold thickness measurements and assessing inter-observer variability among pregnant women who are overweight and obese

**DOI:** 10.1186/1471-2393-13-42

**Published:** 2013-02-19

**Authors:** Lavern M Kannieappan, Andrea R Deussen, Rosalie M Grivell, Lisa Yelland, Jodie M Dodd

**Affiliations:** 1 Discipline of Obstetrics and Gynaecology, School of Paediatrics and Reproductive Health, the University of Adelaide, Adelaide, Australia; 2 The University of Adelaide, Robinson Institute, Discipline of Obstetrics & Gynaecology, Women’s and Children’s Hospital, 72 King William Road, 5006, North Adelaide, South Australia

**Keywords:** Overweight, Obese, Anthropometric measurements, Body composition, Inter-observer variability, Pregnancy

## Abstract

**Background:**

It is estimated that between 34% and 50% of Australian women entering pregnancy are overweight and obese, which is associated with an increased risk in complications for both the woman and her infant. Current tools used in clinical and research practice for measuring body composition include body mass index (BMI), waist circumference and bioimpedance analysis. Not all of these measures are applicable for use during pregnancy due to a lack of differentiation between maternal and fetal contributions. While skinfold thickness measurement (SFTM) is increasingly being used in pregnancy, there is limited data and a lack of a standard tool for its use in overweight and obese pregnant women.

**Methods:**

We developed a standard tool for evaluating SFTM among women with a BMI ≥ 25 kg/m^2^. Forty-nine women were measured as part of a prospective cohort study nested within a multicentre randomised controlled trial (The LIMIT Randomised Controlled Trial). Two blinded observers each performed 2 skinfold measurements on the biceps, triceps and subscapular of each woman. Intraclass correlation coefficients (ICC) and standard error of measurement (SEM) were used to analyse SFTM, body fat percentage (BF%) and inter-observer variability.

**Results:**

The ICC for inter-observer variability in measurements were considered moderate for biceps SFTM (ICC = 0.56) and triceps SFTM (ICC = 0.51); good for subscapular SFTM (ICC = 0.71) and BF% (ICC = 0.74); and excellent for arm circumference (ICC = 0.97). The standard error of measurements ranged from 0.53 cm for arm circumference to 3.58 mm for the subscapular SFTM.

**Conclusion:**

Our findings indicate that arm circumference and biceps, triceps and subscapular SFTM can be reliably obtained from overweight and obese pregnant women to calculate BF%, using multiple observers, and can be used in a research setting.

**Trial registration:**

Australian and New Zealand Clinical Trials Registry ACTRN12607000161426

## Background

Overweight and obesity are significant health problems, and are increasingly encountered during pregnancy and childbirth. It is estimated that between 34% [1] and 50% [2,3] of Australian women enter pregnancy with a body mass index (BMI) greater than or equal to 25 kg/m^2^, placing women and their infants at significant risk of complications [2,4]. Furthermore, the risk of documented complications including pre-eclampsia and gestational diabetes increases with increasing BMI [1,2,4].

A number of tools have been proposed to assess maternal body composition and changes which occur over the course of pregnancy. BMI utilises weight for height measurement (weight in kilograms divided by height in metres squared), and correlates well with indices of adult health [5]. However, it does not take into account the individual components of body composition including adipose tissue and lean muscle mass [5], nor does it reflect changes that occur during pregnancy, including the contribution from products of conception. Similarly, bioimpedance analysis (BIA), while differentiating between lean and adipose tissue mass, does not further differentiate between maternal and fetal contributions [6]. Skinfold thickness measurements (SFTM) have been used to estimate total body fat percentage utilising between three and seven sites across the body [7].

While a number of studies have investigated changes in maternal body composition during pregnancy [8,9], relatively little has been documented about their use in women who are overweight or obese. In particular, these studies have utilised BIA and anthropometric data such as BMI and waist circumference to evaluate body composition [9]. Skinfold thickness measurements have been proposed for use in pregnancy, as they are reproducible with specific training and adherence to defined protocols [7]. Furthermore, use of the biceps, triceps and subscapular sites allow evaluation of pregnancy related changes in adipose tissue that are not influenced by fetal growth.

To our knowledge there is no standard tool in the literature describing the measurement of body composition in overweight and obese pregnant women. Therefore the purpose of this study was to establish a standardised tool for the assessment of skinfold thickness measurements for the purpose of determining body fat percentage, and to evaluate the inter-observer variability in assessing body composition in this group of women.

## Methods

This prospective cohort study is nested within the LIMIT randomised trial, evaluating the effect of an antenatal dietary and lifestyle intervention for women who are overweight or obese. The methodology of the LIMIT randomised trial has been described in detail previously [10].

Women were recruited with a live singleton pregnancy, between 10^+0^ and 20^+0^ weeks’ gestation, at the time of their first antenatal appointment. All women provided written informed consent to participate. Women were recruited from public maternity hospitals across the South Australian metropolitan area (specifically, Women’s and Children’s Hospital, Lyell McEwin Hospital, and Flinders Medical Centre). Ethics approval was obtained from all sites.

At the time of study entry, all women had their height and weight measured, and BMI calculated. Women were then categorised according to their BMI as either overweight (BMI 25.0-29.9 kg/m^2^) or obese (BMI ≥30.0 kg/m^2^), utilising World Health Organisation criteria [5].

### Anthropometric measurements

Skinfold thickness measurements were performed as per the International Standards for Anthropometric Assessment Manual (2006) [7]. All measurements were performed on the right-hand side of the body, unless otherwise stated. After measuring the arm circumference, biceps, triceps and subscapular skinfold thickness measurements were obtained using Harpenden Callipers. Dial graduation of the callipers was 0.2 mm with a measuring range of 0-80 mm. The calliper dial was viewed at 90° to avoid errors of parallax. Two measurements were taken and if the difference was greater than 7.5% a third measurement was performed [7]. The final measurements were recorded to the nearest 0.1 mm and were reported as the average of two measurements or the median of three (if three measurements were obtained).

To measure the arm circumference, the woman was asked to stand with her arms relaxed at her side. The midpoint between the most superior and lateral point of the acromion border and the most proximal and lateral border of the head of the radius was determined [7]. Using the cross hand technique, the arm circumference was measured at this point, ensuring it was taken at eye level, and with constant tension applied to the tape. With the tape still around the midpoint of the arm, a mark was made on the most anterior point of the biceps (just above measuring tape) and the most posterior point of the triceps (just below measuring tape) area to assist in locating the biceps and triceps skinfold landmark [7].

#### Measuring skinfold thickness

To measure skinfold thickness, the indicator on the callipers was zeroed. The thumb and index finger were held parallel and used to grasp the skinfold, ensuring the skin was rolled from side to side to remove any muscle. The callipers were placed at 90° to the skin, one centimetre distal to the marked skinfold site with the measurement taken after two seconds [7].

#### Location of skinfold sites

##### Biceps

The woman was asked to stand relaxed, with arms at her side, and the biceps skinfold was visualised by standing in front of her. The site was located on the anterior surface of the arm, in line with the mid-arm point (as marked when the arm circumference was measured) and parallel to the long axis [7]. Measurement of the biceps and triceps skinfold thickness measurements were alternated to allow tissue decompression.

##### Triceps

To measure the triceps skinfold thickness, the woman was asked to stand relaxed with her right arm slightly pronated. Standing behind her, the triceps skinfold thickness measurement was visualised from the posterior surface of the arm in line with the mid-arm, marked in the horizontal plane of the arm and parallel to the long axis [7].

##### Subscapular

To locate the subscapular skinfold site, the thumb was used to palpate the inferior angle of the scapula, and the site marked 2 cm and 45° inferior to this site the subscapular skinfold being oblique to the landmark. If required, the woman was asked to reach behind her back with her right arm to better expose the scapula [7].

### Body fat percentage (BF%)

To our knowledge, there are no published equations for calculating BF% in women who are overweight or obese using these measurements. The following equation was developed specifically for this study in a sample of 721 women and validated in a sample of 481 women with similar characteristics to women participating in the LIMIT trial:

BF%=12.7+0.457×tricepsSFTM+0.352×subscapularSFTM+0.103×bicepsSFTM−0.057×height+0.265×armcircumference,

Where SFTM were measured in mm, and arm circumference and height were measured in cm (personal communication, Timothy Olds, 15/09/12)

### Inter-observer variability

Dual measurements were collected to assess inter-observer variability. Research assistants had identical training, adhered to the same protocol and were blinded to each other’s measurements. One research assistant completed all measurements (arm circumference and skinfold thickness measurements) on each woman. Landmarks were then removed and a second research assistant repeated the identical procedure on each woman to obtain a second set of measurements straight after the first set of skinfold measurements were completed by the first observer. All measurements were done at single visit at either Trial entry or 36 weeks gestation and the two observers were not necessarily the same for each woman.

### Data analysis

Statistical analyses were performed with the use of SAS software, version 9.2 (Cary, NC, USA). The mean and range of the SFTMs were calculated across all observers and women.

Correlation between anthropometric measures was calculated using intra-class correlation coefficients (ICC) to determine variability between researchers performing maternal body composition measures. A random observer model was used to allow for participation of multiple observers and the standard error of measurement was also obtained from this model. There are no standardised values for accepting reliability when using an ICC but Portney and Watkins [11] suggest a range with values from zero to one, with one indicating perfect agreement. The use of ICC’s to judge validity of a measurement is dependent on the nature of the measurement and what is being described [11]. Portney and Watkins describe an ICC of 0–0.75 as indicating poor to moderate reliability and an ICC of above 0.75 indicating good reliability.

## Results

During the study period, 49 women participating in the LIMIT Study were each assessed by two observers. The mean age of the women was 29.7 years (SD = 5.2 years), with a mean gestational age (GA) at study entry of 13.4 weeks (interquartile range (IQR) 11.3-16.3 weeks). A total of 24 women (49%) were in their first ongoing pregnancy and 6 women were smokers (12.2%). The majority of women (47 women, 95.9%) were of Caucasian ethnicity. The mean BMI at study entry was 29.2 kg/m^2^ (IQR = 27.6-33.6 kg/m^2^) with 27 women (55.1%) classified as overweight (BMI 25.0-29.9 kgm^2^) and 22 women (44.9%) classified as obese (BMI ≥30.0 kg/m^2^) (Table 1).

**Table 1:** Baseline characteristics

**Characteristic**	**n = 49**	General population %*
Maternal Age (Years): Mean (SD)	29.7 (5.2)	No data available
Gestational Age at Entry (Weeks): Median (IQ range)	13.4 (11.3-16.3)	N/A
Parity: 0 (N%)	24 (49.0)	41.5
Public Patient: N (%)	49 (100.0)	74.1
Smoker: N (%)	6 (12.2)	15.9
Ethnicity: Caucasian (N%)	47 (96.0)	85.0
BMI (kg/m^2^): Median (IQ range)	29.2 (27.6-33.6)	No data available
BMI Category (kg/m^2^): N (%)		
BMI 25.0-29.9	27 (55.1)	54.1^#^
BMI 30.0- ≥ 40.0	22 (34.9)	45.9^#^

* Source: Pregnancy Outcome in South Australia 2009, Government of South Australia [3].

# % calculated excluded underweight women and women of normal weight.

*BMI*: Body mass index.

For 49 women, skinfold measurements were taken by 2 observers and the duplicate observations used to calculate the ICC. The ICC for arm circumference was 0.97 indicating excellent reliability. The ICCs for biceps, triceps and subscapular skinfold measurements and BF% were 0.56, 0.50, 0.71 and 0.74 respectively, all indicating moderate reliability (Table 2.) The standard error of measurement for arm circumference, biceps, triceps and subscapular and BF% was 0.53 cm, 2.34 mm, 3.02 mm, 3.58 mm and 2.30% respectively.

**Table 2:** Inter-observer variability of arm circumference and skinfold thickness measurements

**Skinfold Site**	**Average measurement (Range)**	**Standard error of measurement**	**Intraclass correlation coefficient**
Arm Circumference (cm)	34.57 (30.00-46.30)	0.53	0.97
Biceps (mm)	13.21 (6.50-21.30)	2.34	0.56
Triceps (mm)	24.13 (11.10-38.80)	3.02	0.50
Subscapular (mm)	25.37 (11.10-42.30)	3.58	0.71
BF%	33.76 (21.99-47.37)	2.30	0.74

Note: Intraclass correlation coefficient and standard error of measurement were calculated from a one-way random effects model.

## Discussion

To our knowledge, assessment of inter-observer variability in anthropometric measurements in overweight or obese pregnant women has not been characterised previously. This study investigated inter-observer variability in a relatively homogenous population of overweight and obese women who were pregnant and therefore of reproductive age, and were predominantly Caucasian. Further studies may be required to assess inter-observer variability in skinfold measurements taken using the methods we developed in other populations.

The standard error of measurement (SEM) indicates the variability in measurements taken on the same woman by different observers. The biceps skinfold demonstrated the lowest variability from the three skinfold measurements with an SEM of 2.34 mm, while the triceps skinfold had an SEM of 3.02 mm. Although the variability for the subscapular measured was the greatest at 3.58 mm, the correlation between observers using the ICC was the strongest (ICC = 0.71) for this skinfold measurement.

In our study, measurement of the arm circumference demonstrated excellent reliability with an ICC of 0.97. We identified moderate reliability of all skinfold thickness measurements, ranging from an ICC value of 0.50 for the triceps up to 0.71 for the subscapular skinfold measurements. Interpretation of the ICC for BF% is difficult as other studies have not reported this. Our study demonstrated good reliability for BF% (ICC = 0.74), though it should be noted that the ICC for BF% is influenced by the inter-observer variability in the anthropometric measurements used to calculate BF%.

We have identified two small studies [12,13], that have evaluated inter-observer variability in anthropometric measurements. Both studies involved men and non-pregnant women.

Nordhamn and colleagues [12] conducted a prospective cohort study in Sweden to determine the reliability of anthropometric measurements, reporting duplicate measurements from 25 individuals of normal BMI (11 men and 14 non-pregnant women; BMI <26 kg/m^2^) and 26 overweight individuals (13 men and 13 non-pregnant women; BMI ≥26 kg/m^2^). Measurements were taken by two observers on two separate occasions, one to three weeks apart. BMI, waist and hip circumferences and skinfold measurements (biceps, triceps, subscapular, suprailiac, umbilical, anterior thigh, and posterior) were measured and ICCs reported. The ICC for the biceps, triceps and subscapular skinfolds were 0.81, 0.82 and 0.67, respectively. Importantly, in this study, the reliability and inter-observer correlations declined with increasing BMI [12].

Klipstein-Grobusch and colleagues [13] conducted a cohort study in Germany to assess the extent of intra- and inter-observer variability in anthropometric and body composition measurements. Ten healthy volunteers (4 men and 6 non-pregnant women) were measured by seventeen trained observers on two occasions, over a three day period. Measurements included height, weight, sitting height, body circumferences (waist, hip and mid-arm), skinfold thickness measurements (biceps, triceps, subscapular and suprailiac), and chest breadth and depth. In this study, the reported ICC indicated strong correlation with arm circumference (ICC 0.97), biceps (ICC 0.86), triceps (ICC 0.97) and subscapular (ICC 0.94) skinfold thickness measurements [13].

There are key reasons for the differences observed in the current study compared with those reported in the literature [12,13]. While the study by Nordhamn and colleagues included overweight individuals, the study by Klipstein-Grobusch did not. This important difference in study populations could account for differences observed in our study, recognising the difficulty which exists in accurate identification of landmarks and skinfold sites in overweight and obese women. Both studies reported in the literature [12,13] are further limited by their relatively small sample size. Despite these differences, the percentage variability and ICC for arm circumference we obtained were comparable with those reported in the literature, indicating that our methodology and protocol were robust.

Other studies have been identified in the literature reporting inter-observer variability in pregnant women [14-17]. However these studies did not describe in sufficient detail the methodology used to determine this variability [14,15], or used different methodology preventing comparison of results [16,17].

A limitation of this study was that we were not able to use an established method for assessing BF% for comparison with BF% calculated from skinfold thickness measurements. Use of an established method was not feasible for the current study, as most methods for calculating body composition are too expensive (for example Dual energy X-ray absorptiometry, magnetic resonance imaging or computed tomography) or unable to differentiate between maternal and fetal components (BIA and waist circumference) and are therefore not appropriate for use in pregnancy[6].

## Conclusion

We identified excellent correlation for the arm circumference measurement, good correlation for the subscapular SFTM and moderate correlation for both biceps and triceps SFTM. Our study used a larger sample size than has previously been reported and to our knowledge, is the first to report inter-observer variability in anthropometric measures specifically obtained in overweight or obese pregnant women.

Our findings indicate that arm circumference, biceps, triceps and subscapular SFTM can be reliably obtained from overweight and obese pregnant women to calculate BF%, using multiple observers, and can be used in a research setting.

### Ethical approval

Ethics approval was obtained from the Child, Youth, and Women’s Health Service (REC 1839/6/2012, 27/05/2009), Flinders Medical Centre (Application 128/08, 29/05/2008) and Lyell McEwin Hospital (Application Number 2008033, 12/05/2009) research and ethics committees.

## Abbreviations

BMI: Body mass index;ICC: Intraclass coefficient;BIA: Bioimpedance analysis;SFTM: Skinfold thickness measurements;SEM: Standard error of measurement

## Competing interests

The authors declare that they have no competing interests.

## Authors’ contribution

JMD conceived the study design; LMK, ARD and JMD drafted the manuscript; all authors contributed to critical revision of the manuscript drafts and gave final approval for publication.

## Pre-publication history

The pre-publication history for this paper can be accessed here:

http://www.biomedcentral.com/1471-2393/13/42/prepub
